# Atypical presentation of unilateral pulmonary artery agenesis diagnosed postpartum

**DOI:** 10.1016/j.radcr.2024.05.071

**Published:** 2024-06-19

**Authors:** Zekarias Ayalew, Gebeyehu Azibte, Yinager Minaye Anagaw

**Affiliations:** a Department of Internal Medicine, Addis Ababa University, College of Health Sciences, Addis Ababa, Ethiopia; b Department of Radiology, Bahir Dar University, College of Health Sciences, Bahirdar Ethiopia

**Keywords:** UAPA, Congenital condition, PDA, Pregnancy, Postpartum

## Abstract

A 27-year-old woman developed a cough, shortness of breath, and symptoms mimicking pregnancy complications 6 days after childbirth. Unilateral pulmonary artery agenesis (UAPA), a rare congenital condition, was diagnosed through a chest CT scan. This case highlights the variable presentation of UAPA, even in adults, and the challenges of diagnosis during the postpartum period. Early diagnosis and management are critical for improving pregnancy outcomes in women with UAPA.

## Introduction

Unilateral Pulmonary Artery Agenesis (UAPA) is a rare congenital disorder affecting approximately 1 in 200,000 young adults [1]. It typically affects the right pulmonary artery and arises from abnormal aortic arch development. UAPA can occur as an isolated anomaly or, more commonly, with cardiovascular anomalies in two-thirds of patients [2]. Tetralogy of Fallot (TOF), truncus arteriosus, and ventricular septal defect (VSD) are the most frequently associated anomalies. Consequently, these patients often present early in infancy due to cyanosis or symptoms of heart failure, while for patients presenting with isolated UAPA, diagnosing it in infancy is challenging due to the asymptomatic nature of the condition [3]. Those patients presented early during infancy due to cyanosis and symptoms of heart failure [4]. In contrast, isolated UAPA typically remains asymptomatic until adulthood [3]. This case report describes a 27-year-old woman who presented to the emergency department on her sixth postpartum day with a cough and shortness of breath of 3 days' duration. Pulmonary embolism, pregnancy, and pneumonia were initially considered due to her symptoms and the presence of risk factors.

## Case presentation

A 27-year-old woman presented with pleuritic chest pain, shortness of breath during exertion, and dry cough of 3 days duration. She gave birth 6 days back via spontaneous vaginal delivery, and the pregnancy was complicated by preeclampsia with severe features. Otherwise, no fever or hemoptysis. There was no headache, right upper quadrant pain, or personal or family history of cardiac illness in the past. She had 2 previous pregnancy losses in the second trimester.

Physical examination revealed tachycardic (104 bpm), tachypneic (25), afebrile, normal blood pressure (100/70 mmHg), and oxygen saturation of 85% with atmospheric oxygen. The JVP is raised, the chest is clear, and there is grade 1 pitting pretibial edema. Other parts of the physical examinations were unremarkable. Laboratory evaluations, including complete blood count, renal function tests, and liver enzymes, were within the normal reference ranges. A D-dimer test was planned but unavailable due to laboratory limitations.

**Fig. 1:**
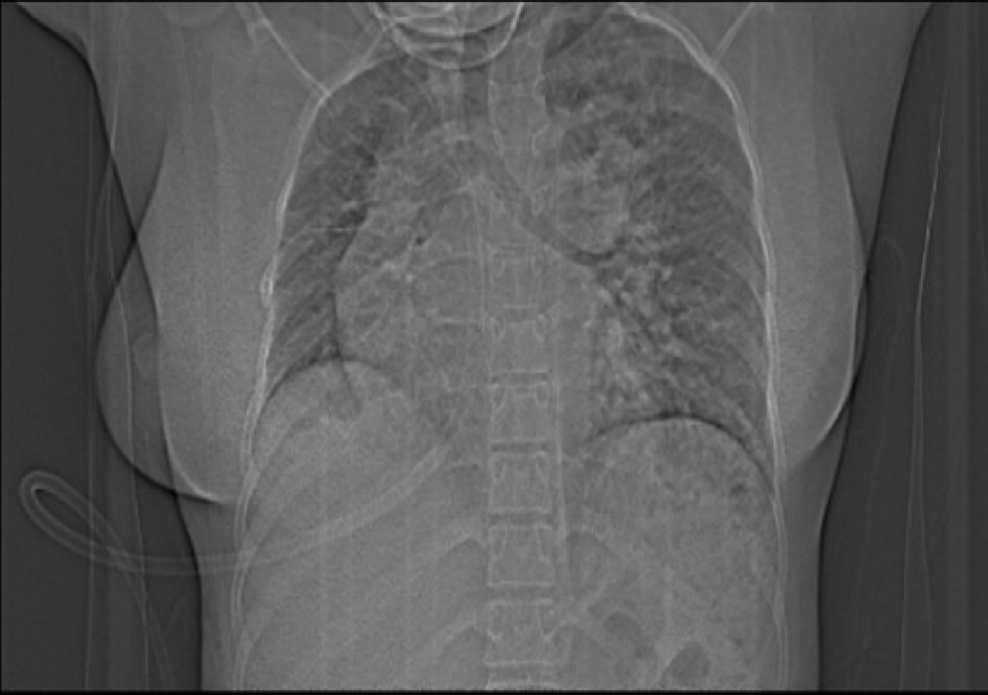
Scout image showing decreased right lung volume and left perihilar and upper lobe air space opacity.

**Fig. 2:**
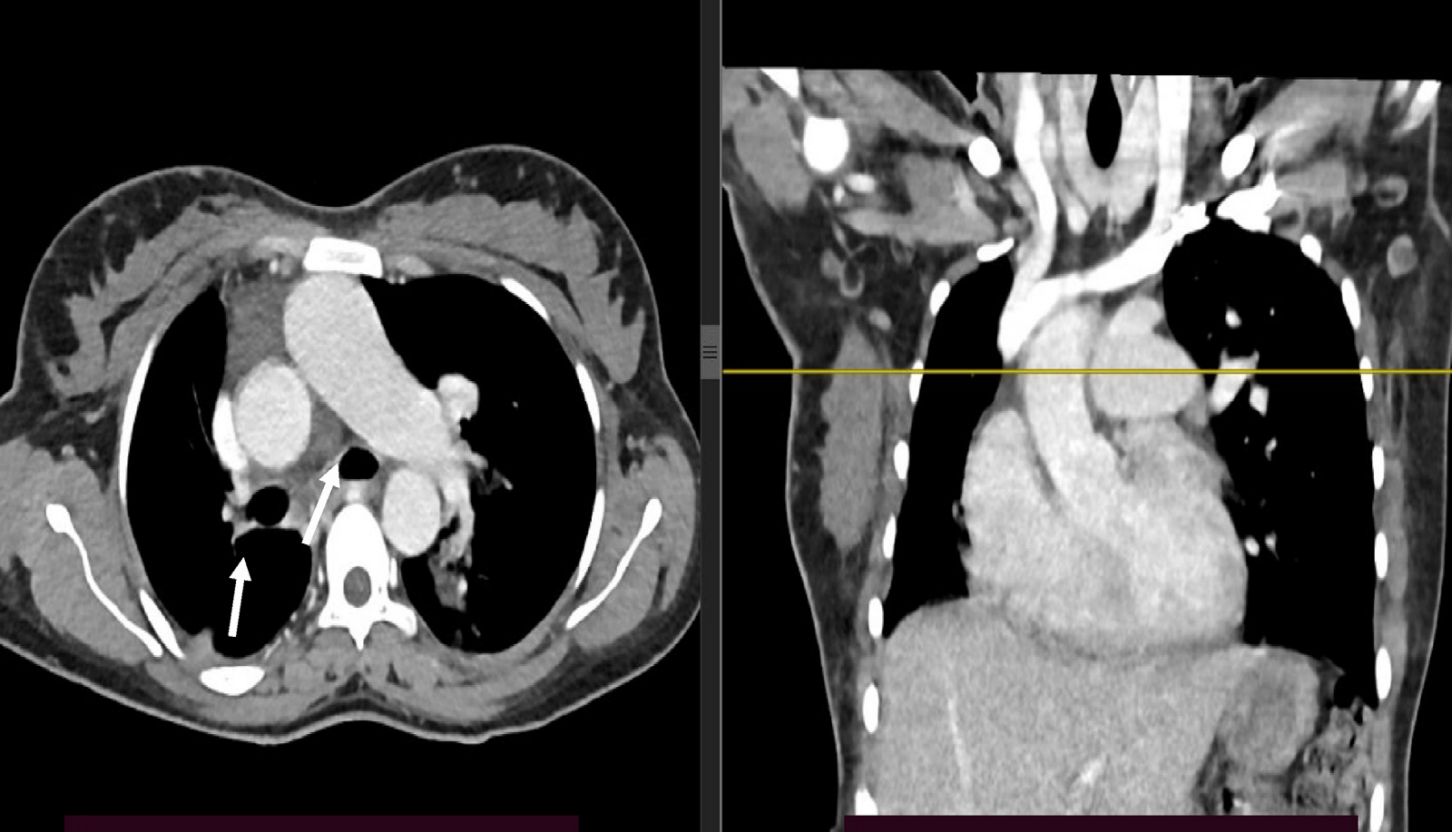
Axial and coronal mediastinal window showing absent right main pulmonary artery, hypoplastic right lung, and its branches, replaced by systemic collaterals.

**Fig. 3:**
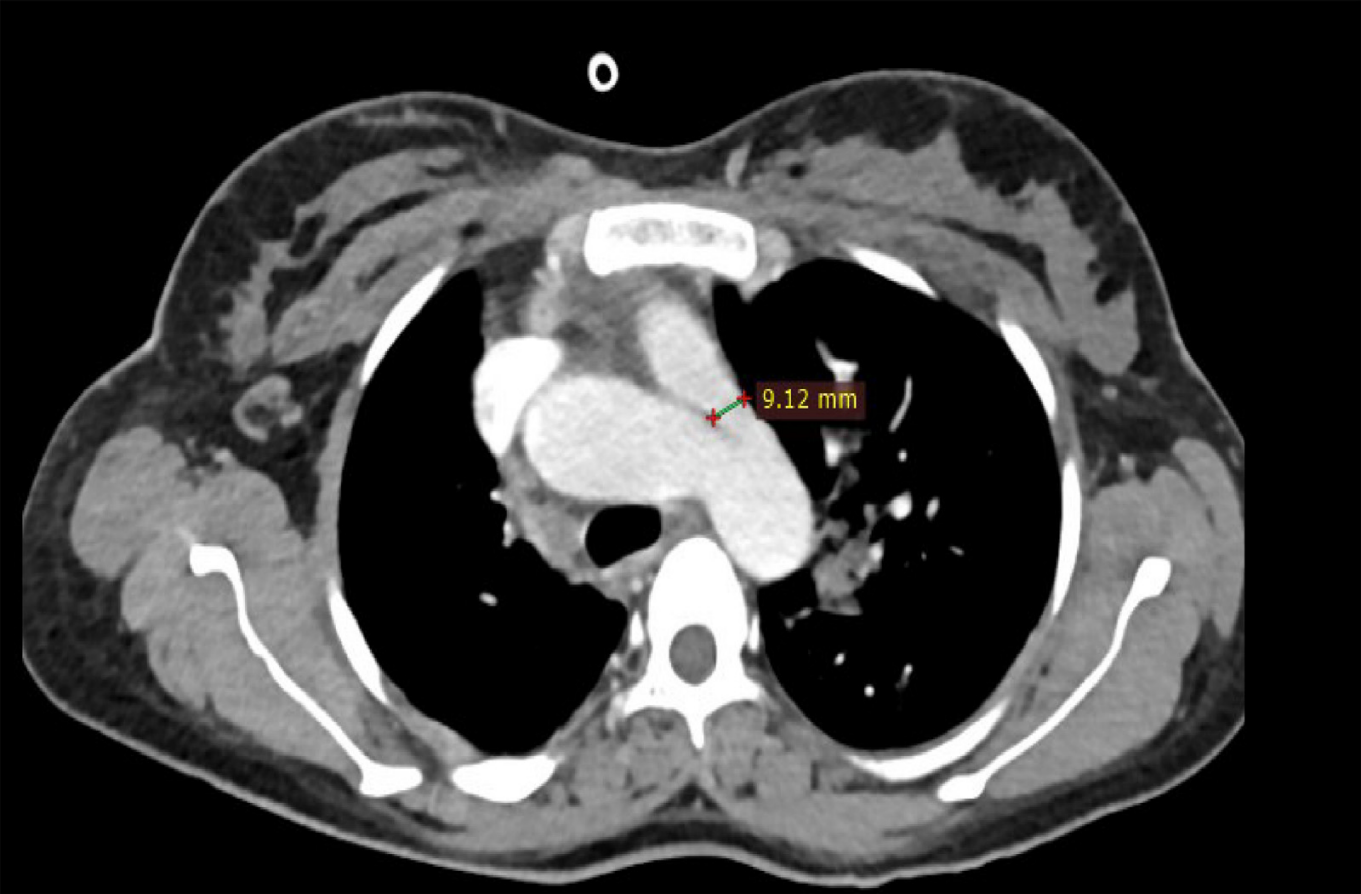
Axial mediastinal window images showing direct communication between the arch of the aorta and the main pulmonary artery by tubular structure.

**Fig. 4:**
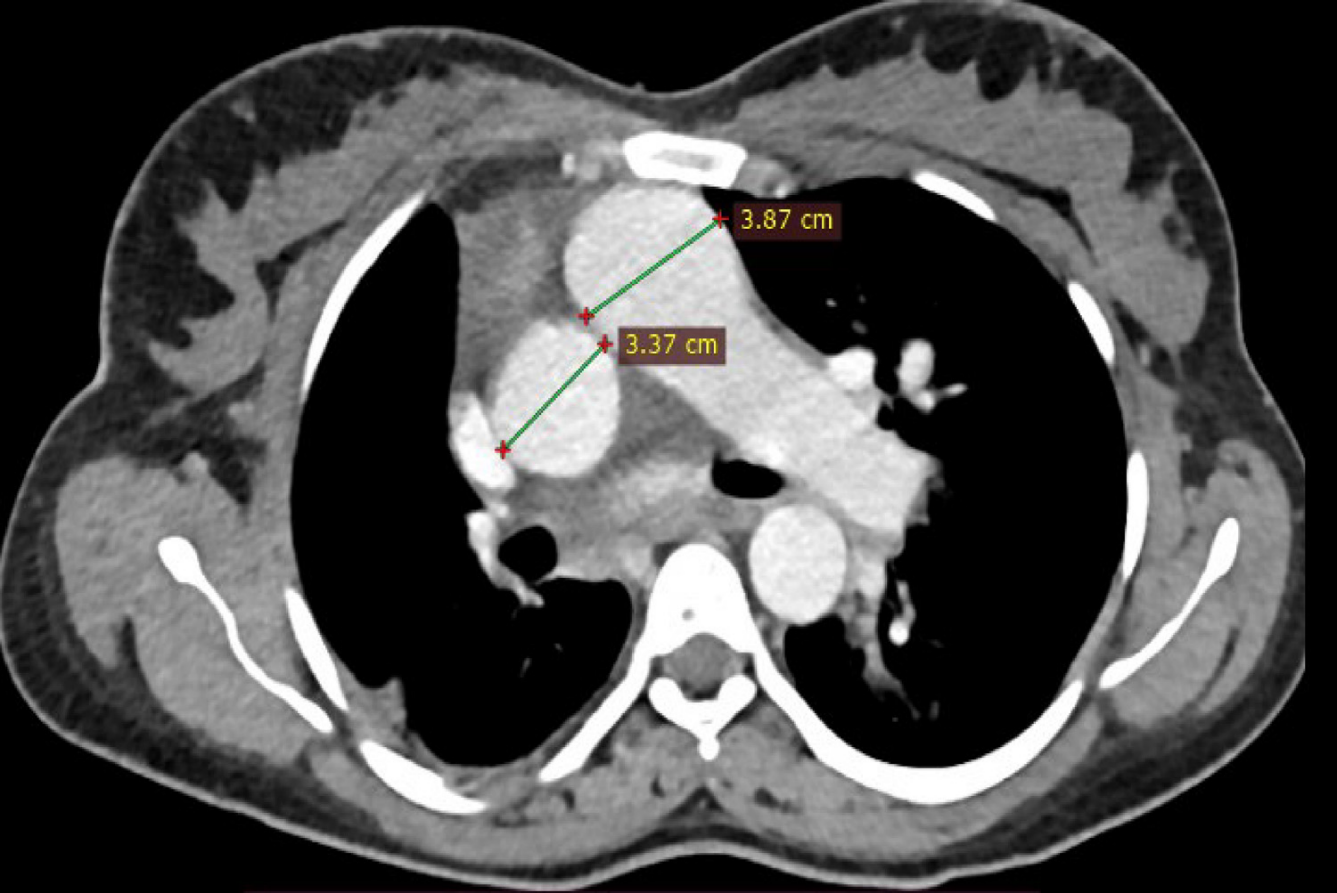
Axial mediastinal Windom images showing enlarged main pulmonary artery.

**Fig. 5:**
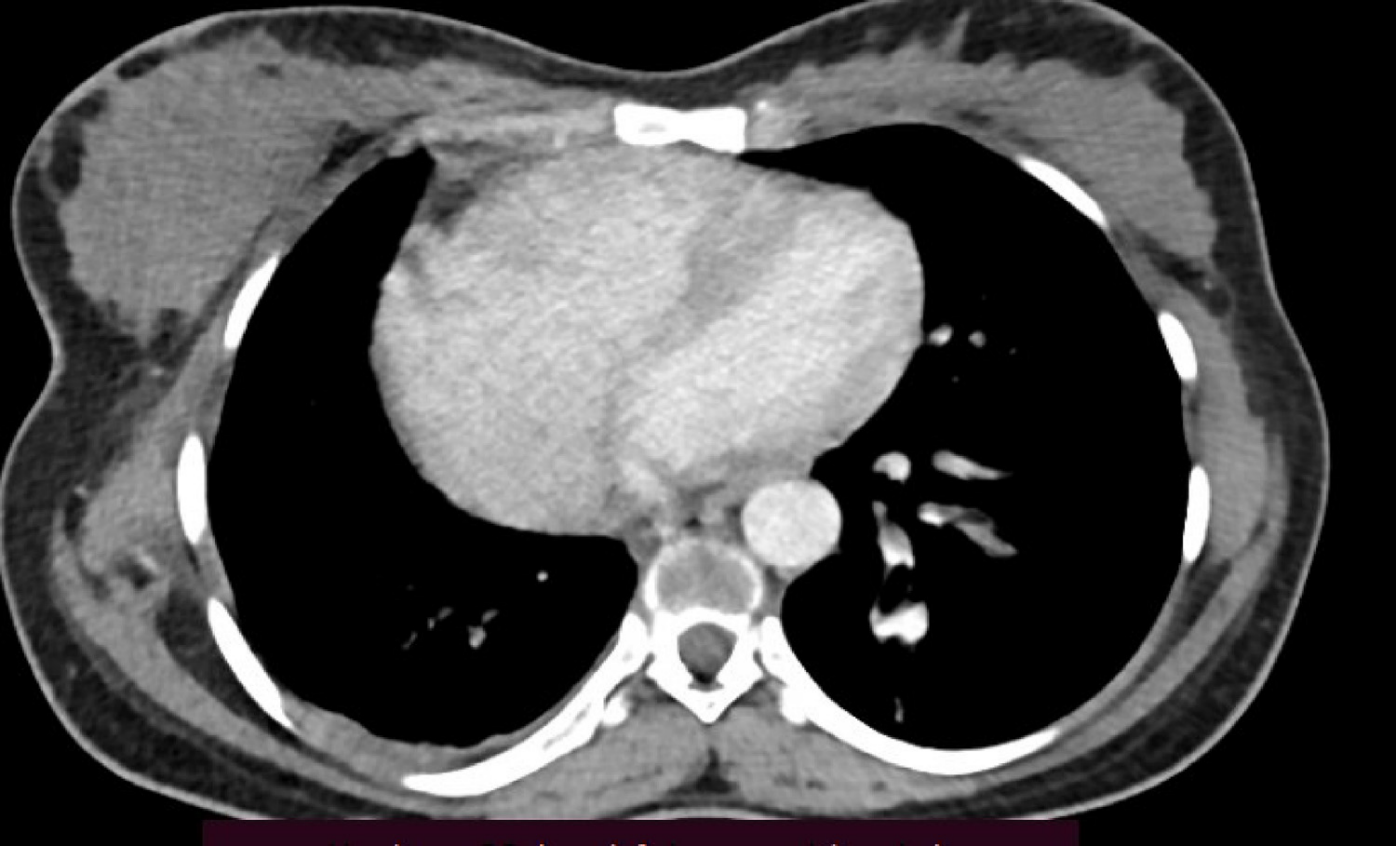
Axial mediastinal Windom images showing enlarged right cardiac chambers with a leftward deviation of the inter-ventricular septum.

**Fig. 6:**
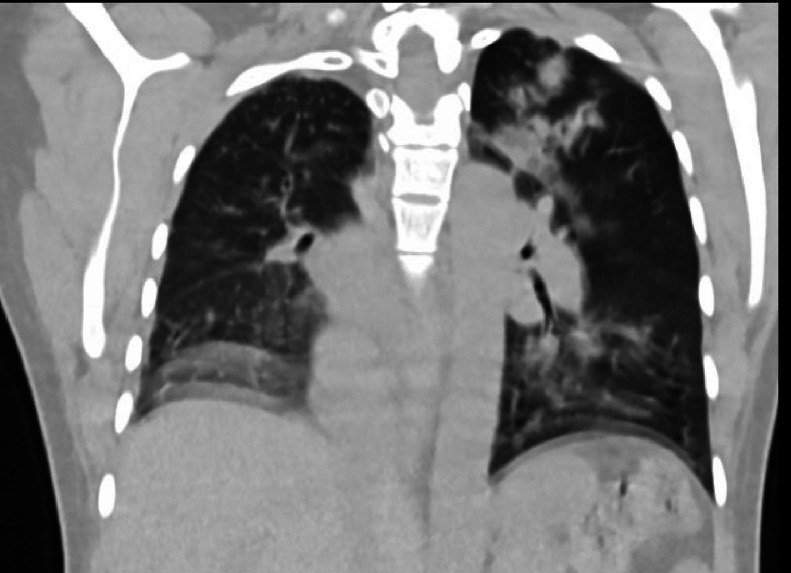
Axial and coronal lung Windom images showing the reduced volume of the right lung with multiple peri hilar and peri broncho vascular patchy air space opacity of the left lung.

Chest CT with contrast demonstrated significant abnormalities. A scout image demonstrates findings suggestive of decreased right lung volume and airspace opacities in the left perihilar and upper lobe regions (Fig. 1). There was complete agenesis of the right main pulmonary artery and its branches with compensatory supply to the hypoplastic right lung arising from anomalous branches of the thoracic aorta and intercostal arteries (Fig. 2). Direct communication was between the main pulmonary artery and the aortic arch (Fig. 3). The main pulmonary artery measured 38 mm, and the left pulmonary artery showed normal anatomy and contrast enhancement (Fig. 4). There was also cardiomegaly (Fig. 5). Lung windows demonstrated a multifocal airspace opacity in the left lung with a peri-hilar and peri-Broncho vascular distribution (Fig. 6). In addition, the echocardiography revealed PDA, cardiomegaly with severe pulmonary hypertension.

### Treatment and outcome

She was put on intranasal oxygen and started on diuresis. Then, her medical condition improved within 3 days of treatment, and she was discharged home with a cardiologist appointment. On her last follow-up 3 weeks back, she complained of exertional shortness of breath. She indicated surgery, but she could not afford the surgery due to financial reasons.

## Discussion

A literature review encompassing 108 cases reported from 1978 to 2000 revealed a median age of diagnosis at 14 years. The most frequent symptoms were dyspnea (40%), frequent pulmonary infections (37%), and hemoptysis (20%). Additionally, around 44% of patients developed pulmonary hypertension [5]. This variability in presentation can lead to misdiagnosis, particularly in adults [6]. Our patient was 27 years old, and her initial presentation was during the postpartum period.

Meta-analysis of UAPA in pregnancy was reported in 2023. It includes a total of 22 pregnancies that were found to have increased adverse pregnancy outcomes with increased preterm and operative deliveries (Cesarian section and/or operative vaginal), and 1 patient had immediate postpartum death. Nearly half (46.2%) had a cardiac malformation, and one-fourth of patients had pulmonary hypertension [7]. Our patient had PDA and pulmonary hypertension. However, the delivery was via SVD, and no complications. After sixth post-partum day, she developed a dry cough, chest pain, and shortness of breath. Her history of preeclampsia with severity features and two episodes of spontaneous second-trimester pregnancy loss made the diagnosis more complex, mimicking pulmonary edema, pulmonary embolism (PE), and pneumonia.

Pulmonary edema was considered because of the history of preeclampsia, but the blood pressure and urine analysis were average. Considering the acute presentation and increased risk of thrombosis during the postpartum period, pulmonary embolism was considered. But there was no imaging evidence. There was 1 report of a coincidence between UAPA and PE [8].

Recurrent infections can be caused by impaired blood flow, leading to impaired delivery of immune cells and bronchoconstriction due to alveolar hypocapnia. This can ultimately result in mucus plugging, chronic bronchitis, and bronchiectasis. Although our patient had no history of recurrent infections, pneumonia was considered a differential diagnosis [[1], [8]].

The diagnosis of UAPA can be done using various imaging modalities, including echocardiography, CT, MRI, and angiography. Typical findings on these imaging studies include the absence of the pulmonary artery, intact peripheral branches of the pulmonary artery, and the presence of collateral vessels. Echocardiography is particularly advantageous as it can detect cardiac anomalies. Though angiography is a gold standard, it is an invasive procedure [[6], [9]]. Our patient's diagnosis was made through a CT scan.

There is no universally agreed-upon treatment for UAPA. The approach is individualized based on the patient's symptoms, the anatomy of the missing pulmonary artery, associated cardiac anatomy, and the presence or absence of Pulmonary hypertension [10]. The treatment options include partial or complete pneumonectomy and revascularization for those patients who had complications such as recurrent infection or hemoptysis and pulmonary hypertension [[11], [12]].

## Conclusion

UAPA presents variably, with atypical presentations during the postpartum period, making diagnosis very challenging. Early diagnosis and appropriate management are crucial for improving pregnancy outcomes in women with UAPA.

## Patient consent

Written informed consent for the publication of this case report was obtained from the patient.
